# Anthropometric parameters as a predictor for abdominal wall thickness in a patient with gastrostomy

**DOI:** 10.1371/journal.pone.0296818

**Published:** 2024-02-23

**Authors:** Prasit Mahawongkajit, Autchariya Saengwijit, Poon Wongvisavavit, Chittinad Havanond, Saritphat Orrapin

**Affiliations:** Department of Surgery, Faculty of Medicine, Thammasat University, Pathumthani, Thailand; AIIMS: All India Institute of Medical Sciences, INDIA

## Abstract

**Background:**

Enteral feeding tubes play essential roles in clinical management and nutritional support. Knowledge of the abdominal wall is beneficial in surgical practice and safe for gastrostomy. Anthropometric parameters are currently used for clinical assessment in many clinical applications. That might be beneficial if we applied anthropometric measurement for thickness prediction of the abdominal wall to the schedule of patients’ gastrostomy care. This study aimed to evaluate the anthropometric parameters of abdominal wall thickness (AWT).

**Methods:**

We conducted a cross-sectional study with anthropometric parameters and CT-measured anterior AWT were assessed and analyzed.

**Results:**

The data are collected from January 2020 to March 2021. Arm circumference and body mass index were strongly correlated with AWT at left upper quadrant area and anterior AWT at middle area. The data was created in an TAWT (Thammasat AWT) chart to represent body parameters to AWT.

**Conclusions:**

Arm circumference is related to AWT. A TAWT chart is designed to help medical personnel evaluate the thickness of the abdominal wall and could guide estimating the gastrostomy tube length.

## Introduction

The knowledge of the layer structure of the abdominal wall is beneficial in surgical practice and permits efficient and safe entry into the peritoneal cavity [1, 2]. Enteral feeding tubes play essential roles in the clinical management and nutritional support of patients with poor voluntary oral in-take, chronic neurological dysphagia, mechanical dysphagia, intestinal failure, cancer, and the critically ill [3–7]. If the gastrointestinal tract is functional, then enteral feeding is usually preferred. Gastrostomy is an accepted option for an enteral feeding tube that could be placed endoscopically, radiologically, or by surgery [6–9].

Anthropometric parameters are methods used for clinical assessment in many clinical situations such as obesity, abdominal incisional hernia, abdominoplasty, and risks for cardiovascular diseases [10–15]. Surgical applications of abdominal wall proficiency might be an essential handling issue for gastrostomy patients. That might be beneficial if we applied physical measurement for the length prediction of the abdominal wall. This study aimed to evaluate the anthropometric parameters of abdominal wall thickness (AWT).

## Material and methods

We conducted a cross-sectional study of adult patients (i.e.,≥15 years) who had been admitted to the Surgical unit of Thammasat University Hospital, a tertiary referral center situated in the northern Bangkok conurbation. The study protocol was approved by the Human Ethics Committee of Thammasat University (Faculty of Medicine); reference no. MTU-EC- SU-1-195/62. The inclusion criteria were the patients with 15–70 years old with general surgical diagnoses. All admitted patients who had elective computed tomography (CT) scans were included and fully informed of the purpose of this study. The CT scan in axial view was measured. The AWT at the upper midline (The landmark is the center point at mid-body in the vertical plane and thoracic spine level 12 in the horizontal plane) and left upper quadrant (The landmark is the center point between mid-body and left frank in the vertical plane at thoracic spine level 12 in the horizontal plane) is measured from the skin to parietal peritoneum with the plain CT scan abdomen that the patient had previously performed. Patients with abnormal abdomen/neck/arm anatomy, previous abdominal surgery including gastrostomy, who performed CT scans over 30 days, emergency cases, and significant weight loss (the loss of >5% of one’s body weight over a period of 6–12 months.) were excluded from the study. Anthropometric parameters were measured; arm circumference (AC), arm thickness (AT), waist circumference (WC), waist thickness (WT), and neck circumference (NC). AC is measured at the midpoint between the head of the humerus to the olecranon, AT is measured with a triceps skinfold caliper at the same point as AC, WC is measured at the umbilicus, WT is measured with a skinfold caliper at the anterior mid-lateral part same point of WC and NC is measured when the patient is in standard Frankfort horizontal position middle of the neck between collarbone and chin.

All anthropometric parameters and CT-measured anterior AWT were assessed in both genders. A normal distribution group performed mean and standard deviation (SD) with Student t-test analysis. A non-parametric test was performed by Mann-Whitney test and reported in the median with interquartile range (iqr).

The relation between anthropometric parameters and CT-measured anterior AWT was assessed by Pearson correlation analysis. The Spearman rank correlation coefficient assessed the non-parametric or non-bivariate normality. The equality of distribution (for more than 50 cases) and bivariate normality were evaluated by the Kolmogorov–Smirnov (K-S) test and Doornik–Hansen omnibus tests, respectively. In the subgroup of subjects by gender, the correlation between anthropometric parameters and CT-measured anterior AWT was assessed by Pearson correlation analysis or Spearman Rank correlation coefficient depending on the equality of distribution and the bivariate normality [16].

The linear relation between anthropometric parameters and anterior AWT was demonstrated in scatter plots that used Cartesian coordinates to illustrate data for anthropometric parameters and anterior AWT.

**Fig 1:**
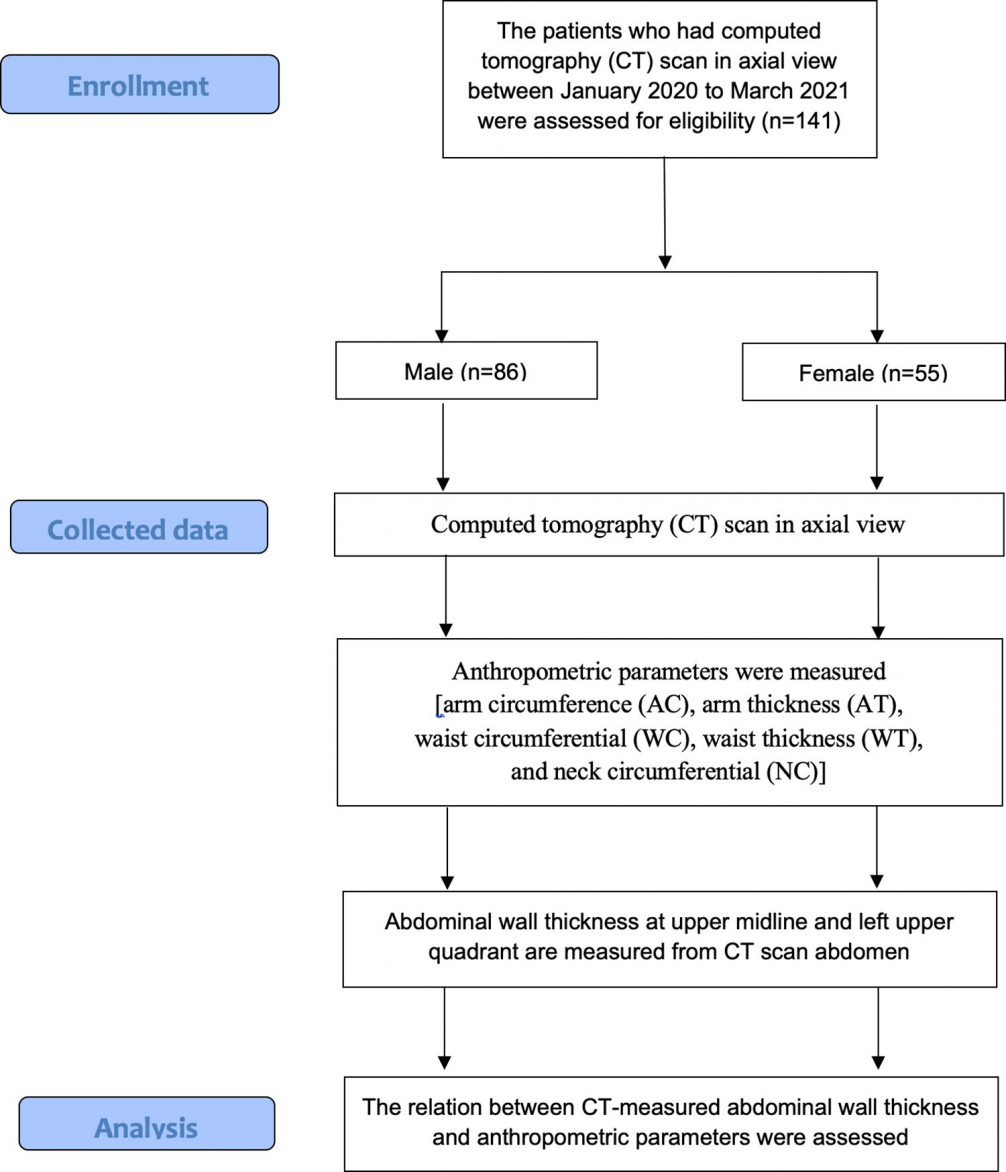
The study flow diagram of cross-sectional study.

All statistical analyses were performed using a STATA/SE 16.0 for Windows (Stata Corp LP, TX, USA), and p-values < 0.05 were regarded as indicating statistical significance. The study process and report followed the strengthening of the reporting of observational studies in epidemiology (STROBE) statement in reports of cohort studies (Fig 1).

## Results

The data are collected from January 2020 to March 2021. Total 141 patients were assessed for eligibility. Baseline characteristic and demographic data were shown in **Table 1.** The anthropometric parameters were collected; AC, AT, WC, WT, and NC with CT-measured anterior AWT at left upper quadrant area (AALUQ).

**Table 1:** Patient characteristics with CT-measured anterior AWT and anthropometric parameters.

Variables	Male (n = 86)	Female (n = 55)	Total (N = 141)	*P-value*
**Age (years) mean ± SD**	61.18 ± 13.6	62.87 ± 16.9	61.84 ± 14.9	0.516
**Body weight (kg) mean ± SD**	59.63 ±13.2	53.87 ± 15.2	57.38 ± 14.2	0.019
**Height (cm) mean ± SD**	163.97 ± 6.8	154.58 ± 8.1	160.31 ± 8.6	< 0.001
**BMI (kg/m^2^) mean ± SD**	21.9 ± 5.0	22.4 ± 5.5	22.11 ± 5.2	0.551
**Arm circumference (cm)**				0.606*
**mean ± SD**	25.69 ± 4.3	25.58 ± 4.7	25.65 ± 4.5	
**median (iqr)**	26 (23,28)	25 (22,28)	26 (22,28)	
**Arm thickness (cm)**				0.092*
**mean ± SD**	0.49 ± 0.3	0.67 ± 0.51	0.56 ± 0.4	
**median (iqr)**	0.4 (0.2,0.8)	0.6 (0.2,1)	0.2 (0.4,0.8)	
**Waist circumference (mm) mean ± SD**	81.84 ± 15.2	82.14 ± 11.8	81.96 ± 14.0	0.901
**Waist thickness (cm)**				0.493*
**mean ± SD**	0.79 ± 0.4	0.87 ± 0.5	0.82 ± 0.5	
**median (iqr)**	0.6 (0.4,1)	0.8 (0.4,1.2)	0.6 (0.4,1.2)	
**Neck circumference (cm) (mean ± SD)**	36.16 ± 4.4	33.2 ± 3.3	34.42 ± 4.1	0.008
**Anterior AWT (mm)**				
**Left upper quadrant**				0.018
**mean ± SD**	17.11 ± 7.3	20.84 ± 9.5	18.56 ± 8.4	
**median (iqr)**	16 (12.3,20)	18.9 (13.3,28)	16.5 (12.8,22.4)	
**Mid abdomen**				< 0.001
**mean ± SD**	12.13 ± 9.7	17.35 ± 8.5	14.17 ± 9.6	
**median (iqr)**	10.4 (6.8,14.15)	16.4 (10.4,24.9)	11.5 (7.5,17.9)	

BMI, body mass index; cm, centimeters; mm, millimeters; iqr, interquartile range; kg, kilogram; m, meters; SD, standard deviation

*Distribution curve and one-sample Kolmogorov-Smirnov test against theoretical normal distribution, the Mann-Whitney test for non-parametric test.

After analysis, the relation between anthropometric parameters and CT-measured AALUQ, we found that AC and body mass index (BMI) were strong monotonic correlation (Spearman correlation coefficient; r_s_ = 0.60–0.79) with AALUQ thickness and other parameters are moderate and weak correlation that demonstrated in **Table 2** (16). AC and BMI were strong correlated with anterior AWT at middle area (AAMID) thickness as exhibited in **Table 3.** The difference between gender as indicated in **Table 4.** The study found that anthropometric parameters are correlated to CT AWT in female group more than male especially in LUQ thickness.

**Table 2:** The relation between anthropometric parameters and CT-measured anterior AWT at left upper quadrant area (AALUQ).

Anthropometric parameters related with AALUQ thickness	Correlation coefficient (Spearman’s rho (r_s_))	*P-value*	Effect Size of correlation
**Age (years) (mean ± SD)**	0.001**	1.000	very weak
**Body weight (kg) (mean ± SD)**	0.569**	< 0.001	moderate
**Height (cm) (mean ± SD)**	-0.238**	0.195	weak
**BMI (kg/m^2^) (mean ± SD)**	0.608**	< 0.001	strong
**Arm circumferential (cm)**	0.669*,**	< 0.001	strong
**Arm thickness (cm)**	0.529*,**	< 0.001	moderate
**Waist circumferential (mm) (mean ± SD)**	0.412**	< 0.001	moderate
**Waist thickness (cm) (mean ± SD)**	0.519*,**	< 0.001	moderate
**Neck circumferential (cm) (mean ± SD)**	0.359**	< 0.001	moderate

AALUQ, anterior abdominal wall at left upper quadrant area; BMI, body mass index; CI, confidence interval; cm, centimeters; mm, millimeters; iqr, interquartile range; kg, kilogram; m, meters; SD, standard deviation

*One-sample Kolmogorov-Smirnov test against theoretical normal distribution, the Spearman Rank correlation coefficient test for non-parametric test.

**Against the bivariate normality by Doornik–Hansen omnibus tests, the Spearman Rank correlation coefficient test was assessed.

**Table 3:** The relation between anthropometric parameters and CT-measured anterior AWT at middle area (AAMID).

Anthropometric parameters related with AAMID thickness	Correlation coefficient (Spearman’s rho (r_s_))	*P-value*	Effect Size of correlation
**Age (years) (mean ± SD)**	0.001**	1.000	very weak
**Body weight (kg) (mean ± SD)**	0.459**	< 0.001	moderate
**Height (cm) (mean ± SD)**	-0.238**	0.195	weak
**BMI (kg/m^2^) (mean ± SD)**	0.608**	< 0.001	strong
**Arm circumferential (cm)**	0.569*,**	< 0.001	moderate
**Arm thickness (cm)**	0.529*,**	< 0.001	moderate
**Waist circumferential (mm) (mean ± SD)**	0.412**	< 0.001	moderate
**Waist thickness (cm) (mean ± SD)**	0.519*,**	< 0.001	moderate
**Neck circumferential (cm) (mean ± SD)**	0.359**	< 0.001	moderate

AAMID, anterior abdominal wall at middle area; BMI, body mass index; CI, confidence interval; cm, centimeters; mm, millimeters; iqr, interquartile range; kg, kilogram; m, meters; SD, standard deviation

*One-sample Kolmogorov-Smirnov test against theoretical normal distribution, the Spearman Rank correlation coefficient test for non-parametric test.

**Against the bivariate normality by Doornik–Hansen omnibus tests, the Spearman Rank correlation coefficient test was assessed.

**Table 4:** The relation between anthropometric parameters and CT-measured anterior AWT at left upper quadrant area (AALUQ) and middle area (AAMID) by gender.

Anthropometric parameters related with AWT	Correlation coefficient (Spearman’s rho (r_s_))		*P-value*		Effect Size of correlation	
	*male*	*female*	*male*	*female*	*male*	*female*
**Age (years)**						
• **AALUQ**	-0.002**	0.010**	1.000	1.000	very weak	very weak
• **AAMID**	-0.066**	-0.086**	1.000	1.000	very weak	very weak
**Body weight (kg)**						
• **AALUQ**	0.573*,**	0.713*,**	< 0.001	< 0.001	moderate	strong
• **AAMID**	0.595**	0.670**	< 0.001	< 0.001	moderate	strong
**Height (cm)**						
• **AALUQ**	-0.152**	-0.043**	1.000	1.000	very weak	very weak
• **AAMID**	0.045**	-0.135**	1.000	1.000	very weak	very weak
**BMI (kg/m2)**						
• **AALUQ**	0.706**	0.798**	< 0.001	< 0.001	strong	strong
• **AAMID**	0.586**	0.793**	< 0.001	< 0.001	moderate	strong
**Arm circumferential (cm)**						
• **AALUQ**	0.632**	0.722**	< 0.001	< 0.001	strong	strong
• **AAMID**	0.571*,**	0.733*,**	< 0.001	< 0.001	moderate	strong
**Arm thickness (cm)**						
• **AALUQ**	0.473*,**	0.595*,**	< 0.001	< 0.001	moderate	moderate
• **AAMID**	0.423*,**	0.655*,**	0.002	< 0.001	moderate	strong
**Waist circumferential (mm)**						
• **AALUQ**	0.489**	0.634**	< 0.001	< 0.001	moderate	strong
• **AAMID**	0.358**	0.581**	0.031	< 0.001	weak	moderate
**Waist thickness (cm)**						
• **AALUQ**	0.517*,**	0.530*,**	< 0.001	0.001	moderate	moderate
• **AAMID**	0.522*,**	0.551*,**	< 0.001	< 0.001	moderate	moderate
**Neck circumferential (cm)**						
• **AALUQ**	0.485**	0.628**	< 0.001	< 0.001	moderate	strong
• **AAMID**	0.491**	0.564**	< 0.001	< 0.001	moderate	moderate

AALUQ, anterior abdominal wall at left upper quadrant area; AAMID, anterior abdominal wall at middle area; BMI, body mass index; CI, confidence interval; cm, centimeters; iqr, interquartile range; kg, kilogram; m, meters; SD, standard deviation

*One-sample Kolmogorov-Smirnov test against theoretical normal distribution, the Spearman Rank correlation coefficient test for non-parametric test.

**Against the bivariate normality by Doornik–Hansen omnibus tests, the Spearman Rank correlation coefficient test was assessed.

## Discussion

Gastrostomy is one of the worldwide applications for enteral feeding to the stomach at the left upper quadrant of the abdominal wall. The anatomy of the abdomen demonstrated the critical know-how for the surgical technique to be successful in the procedure. In addition, some patient still requires gastrostomy as the long-term enteral feeding tube, which must schedule for tube changing. The patient nutritional status is dynamic, which creates the variation in body appearance and abdominal wall. It would affect the feeding tube length and replacement, which might be very important to the outcome of nutritional support via the enteral feeding tube. Inappropriate tube length estimation lead to the procedure’s adverse events such as tube dislodgement and buried bumper syndrome [3, 6, 7].

Anthropometric measurement is the favored method for guiding and predicting the current physical condition of a patient. Not only in clinical practice but NC is also used widely in representing half of WC for pants shopping in the Thai tradition. It might be profitable for clinical application if the anthropometric parameters could be instructed on the AWT.

According to the results of this study, some anthropometric parameters are correlated with AWT. BMI and arm circumferential strongly associated with AALUQ. Body weight, arm thickness, waist circumferential, waist thickness and neck circumferential are moderately associated with AALUQ. For AAMID, only BMI demonstrate the strong association to AWT. Body weight, arm circumferential, arm thickness, waist circumferential, waist thickness and neck circumferential are moderately associated with AALUQ. Age and height show weak to very weak association to AALUQ and AAMID. Waist circumferential and thickness and neck circumferential are poorly associated with AWT due to the variation of body habitus and muscle mass which not direct variation to the fat thickness of abdominal wall.

Costa-Ferreira A, et al published the morphometric study of lower abdominal wall which demonstrate the association between the BMI and lower AWT. Moreover, the trilaminar structure with superficial compartment are the dominant part of AWT. The deep fat compartment has a minor contribution to AWT [17]. However the distribution of compartment between upper and lower abdomen be different. The component of superficial fat compartment is higher in lower abdomen which may not well applied to our study. James MK, et al reported the association between low AWT and percutaneous endoscopic gastrostomy complications [18]. Concordant to our research, the BMI and body weight were direct variation to AWT. The preoperative measurement of AWT by pre-procedural imaging can potentially be used to predict the risk of post-PEG complications. The low anterior AWT was associated with higher number of tube dislodgement patients (AWT 18.1 mm vs 26.8 mm for tube dislodgement group and non-tube dislodgement group, respectively; P-value = 0.043). In addition, low BMI was associated with higher number of tube dislodgement patients (BMI 20.1 kg/m^2^ vs 26.9 kg/m^2^ for tube dislodgement group and non-tube dislodgement group, respectively; P-value = 0.043) [18]. However, the mean and the range of AWT, BMI and body weight of the study are higher in our result due to main participant are different between the Caucasian of Jamaica Hospital Medical Center, United States of America and Asian population of Thammasat University, Thailand.

Frank K, et al. demonstrated the influences of age, gender, and BMI on the thickness of the abdominal fatty layers [19]. The study reported the increase in BMI was associated with an increase in total AWT which concordant to our study result. In addition, an increase in age was not associated with total AWT but demonstrated a decrease in thickness of the superficial fatty layer with an increase in thickness of the deep fatty layer. No influence of gender was detected for either the superficial (*P* = 0.255) or the deep (*P* = 0.725) fatty layer which discordant to our result that show higher AALUQ and AAMID in female gender significantly [19].

**Fig 2:**
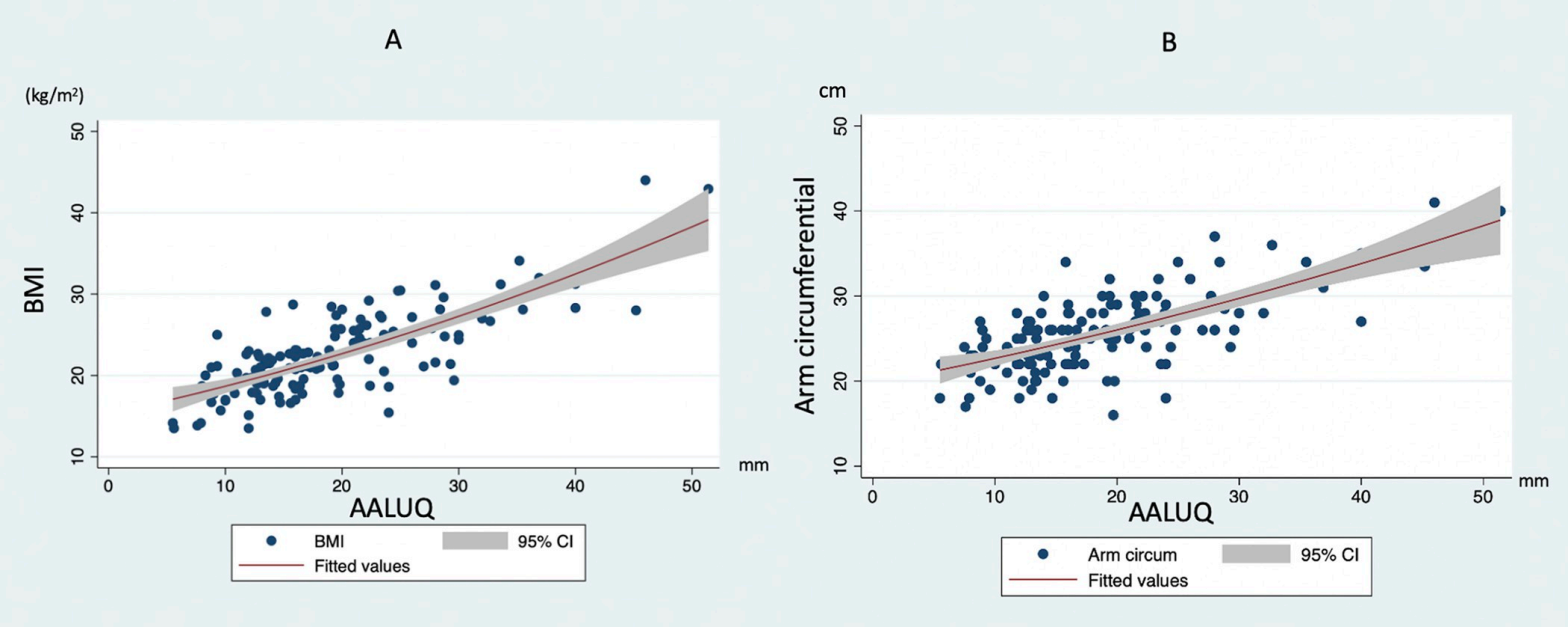
Scatter plot with fitted values and 95% confidence interval between AWT and BMI (A) and Arm circumference (B). Vertical axis of this chart is BMI (kg/m^2^) [left] and Arm circumference (cm) [right] while horizontal axis represents anterior abdominal wall at left upper quadrant area (AALUQ) thickness (mm).

AC and BMI are strongly correlated with AALUQ and AAMID thickness without gender effect (Fig 2). So, our study found that both AC and BMI might be assumed to be a surrogate for the AWT in both male and female genders. However, females are more likely to develop subcutaneous fat due to three shreds of evidence that in vivo catecholamine mediated leg free fatty acid release is lower in women than in men, free fatty acid release by the upper body subcutaneous fat depots is higher in men than in women. Basal fat oxidation (adjusted for fat-free mass) is lower in females than males [20]. For this reason, BMI is in the same range between the two genders so they can represent a correlation to our study. The body weight, waist circumferential which the female gender increases the strength of correlation for AALUQ and AAMID. Abdominal wall layers are composed of multiple layers of skin, subcutaneous fat, and muscles due to the hormonal effect we mentioned earlier.

**Fig 3:**
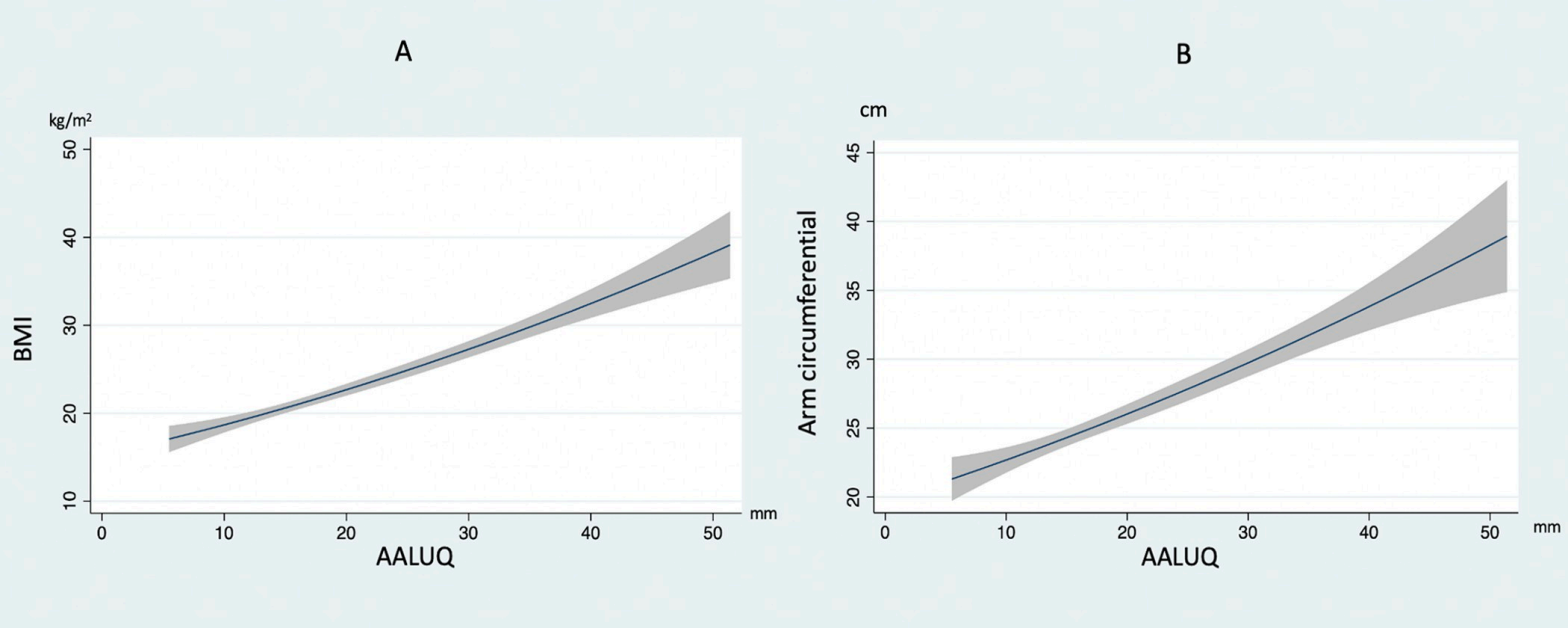
TAWT (Thammasat AWT) chart for predict AWT from BMI (A) and Arm circumference (B). Vertical axis of this chart is BMI (kg/m^2^) [left] and Arm circumference (cm) [right] while horizontal axis represents AWT (mm).

So, we created a TAWT (Thammasat AWT) chart using the quadratic prediction with 95% confidence interval to represent the non-parametric data of the body parameters to AWT (Fig 3). BMI (kg/m2) and AC (cm) best describe AWT due to their strong monotonic correlation in the vertical axis. The horizontal axis is AWT (mm). However, this study had some limitations. One of them lacks previous data, so we cannot estimate the actual sample size. Almost all populations in this study have an average BMI of 22.2 (95% CI 21.45–23.10). The result may be inaccurate if we apply it to the extreme underweight or obese person. Further studies across multiple centers with a large patient number are required and would benefit the knowledge about the anthropometric parameters of AWT. In addition, the external validation of the TAWT chart could be a potential next step before generalized this tool to all patients.

This study demonstrated AC is related to AWT. A TAWT chart is designed to help medical personnel evaluate the thickness of the abdominal wall. Still, the accuracy increases when applied to a person with average body status. This chart is worthwhile because it could guide estimating the gastrostomy tube length and might reduce complications from blind insertion of instruments and techniques.

## Abbreviations

CT: computed tomography
AC: arm circumference
AT: arm thickness
WC: waist circumference
WT: waist thickness
NC: neck circumference
BMI: body mass index
SD: standard deviation
nm: millimeters
cm: centimeters
iqr: interquartile range
AALUQ: AWT at left upper quadrant area
AAMID: anterior AWT at middle area
